# Long-term outcomes at 24- and 36-month follow-up in the intervention arm of the randomized controlled trial of Prompt Mental Health Care

**DOI:** 10.1186/s12888-022-04227-0

**Published:** 2022-09-09

**Authors:** Otto R. F. Smith, Solbjørg M. M. Sæther, Ellen Haug, Marit Knapstad

**Affiliations:** 1 Division of Mental and Physical Health, Department of Health Promotion, Norwegian Institute of Public Health, Zander Kaaes gate 7, 5015 Bergen, Norway; 2 Centre for Evaluation of Public Health Measure, Norwegian Institute of Public Health, Bergen, Norway; 3 Department of Teacher Education, NLA University College, Pb 74 Sandviken, 5812 Bergen, Norway; 4 Department of Health Promotion and Development, University of Bergen, 5020 Bergen, Norway

**Keywords:** Prompt Mental Health Care, Depression, Anxiety, CBT, IAPT, Long-term outcomes

## Abstract

**Background:**

Whether long-term symptom improvement is maintained after treatment in services such as the Norwegian Prompt Mental Health Care (PMHC) and the English Improving Access to Psychological Therapies is not yet known. In this prospective study, we investigate whether improvements observed at 6-month follow-up are maintained at 24- and 36-month follow-up among clients who received PMHC.

**Method:**

Data from the treatment arm of the randomized controlled trial of PMHC were used (*n* = 459). The main outcomes were (reliable) recovery rate and symptoms of depression (PHQ-9) and anxiety (GAD-7). Primary outcome data at 24- and 36-months follow-up were available for 47% and 39% of participants, respectively. Secondary outcomes were work participation, functional status, health-related quality of life, and positive mental well-being. Sensitivity analyses with regard to missing data assumptions were conducted for the primary continuous outcomes.

**Results:**

Improvements were maintained at 24- and 36-month follow-up for symptoms of depression and anxiety, (reliable) recovery rate, and health-related quality of life. Small linear improvements since 6-month follow-up were observed for work participation, functional status, and positive mental well-being. Sensitivity analyses did not substantially alter the findings for symptoms of depression and anxiety mentioned above.

**Conclusions:**

Our findings support the long-term effectiveness of PMHC, but results should be interpreted with caution due to lacking follow-up data at 24- and 36-month in the control group, and substantial attrition.

## Introduction

Cognitive behavioural therapy (CBT) is effective in treating depression and anxiety [1–8]. However, short-term care might not always be enough to sustain improvement over time [9–11]. Individuals with depression [11–15] or anxiety [16] can experience relapse after treatment. For instance, after receiving acute phase CBT for depression, around 29% have been found to experience relapse or recurrence during the first year and 54% during the second year [15]. Programs aiming at alleviating symptoms of depression and anxiety should therefore be thoroughly evaluated – also after end of care.

The service Prompt Mental Health Care (PMHC) was initiated in order to improve access to evidence-based primary care treatment for individuals with symptoms of mild to moderate depression and anxiety disorders in Norway [17, 18]. PMHC is an adapted version of the British Improving Access to Psychological Therapies (IAPT) [19, 20]). It was commissioned by the Norwegian Ministry of Health and Care in 2012 [17] and is today employed in around 70 Norwegian municipalities [21]. PMHC aims to supplement existing services and be low threshold, without need for referral, with short waiting times, and free of charge [17, 22]. All care in PMHC is based on CBT [17, 22], and both low and high-intensity care is offered, in stepped care variants [17, 22].

Research has shown that IAPT treatment is associated with substantial symptom reduction [23, 24]. However, a recent study showed that relapse within the first year after receiving low intensity CBT in IAPT is common (53%) [25]. Clients are seldom followed to determine longer-term outcome after IAPT care [26], and a recent meta-analysis of IAPT studies underlines the importance of further investigating the durability of benefits gained by IAPT interventions [23]. For PMHC, improvements have earlier been found at post-treatment and at 12-month follow-up in observational studies [27–29]. Results from a later randomized controlled trial of PMHC versus treatment as usual (TAU) indicated substantial treatment effects on symptoms of depression and anxiety, (reliable) recovery rate, functional status, health-related quality of life, and positive mental well-being at 6-month follow-up [30]. These treatment effects were maintained at 12-month follow-up [31]. No effect was found on self-reported work participation compared to TAU, and some clients experienced relapse after end of care (at 12 months follow-up, 10% of PMHC clients had relapsed, against 16% in treatment as usual (TAU) [31]. How outcomes evolve in PMHC beyond 12 months is yet to be investigated.

The aim of the present study was therefore to determine whether the observed improvement at 6-month follow-up was maintained at 24- and 36-month follow-up with regard to primary and secondary outcomes for clients who were assigned to the PMHC treatment condition. Sensitivity analyses were conducted in order to explore the impact of missing data assumptions on results for continuous primary outcomes in the presence of attrition.

## Methods

The current study uses primarily data from the treatment arm of an RCT (ClinicalTrials.gov: NCT03238872, registration date: 03/08/2017) comparing PMHC to TAU [30]. The RCT was conducted within routine care in two Norwegian municipalities; Kristiansand and Sandnes, from 2015 to 2017. Details about the trial design are provided in the primary evaluation of the RCT [32].

### Participants

Eligibility for the PMHC service is based on a defined set of inclusion and exclusion criteria, which were also applied in the trial [30]. The main inclusion criteria were anxiety and/or mild to moderate depression (defined as Generalized Anxiety Disorder scale (GAD-7) and/or Patient Health Questionnaire (PHQ-9) scores above cut-off), ≥ 18 years of age, living in Kristiansand or Sandnes, and basic Norwegian language proficiency [30].

All clients contacting PMHC in Sandnes and Kristiansand got an appointment for individual assessment at the PMHC clinic. As described in our earlier work [30], the therapist examined the relevance and severity of the mental health problems, the available client resources, and motivation for treatment. Information about the study and the treatment methodology within PMHC was provided to the client. All information was then reviewed by the therapist upon which the decision on inclusion/exclusion was made, always in consultation with the client [30].

Clients who agreed to participate were asked to register to a secure online data portal specifically developed for the evaluation of PMHC by the Norwegian Centre for Research Data (NSD). When registered, the participants filled in the baseline questionnaire. Following completion, the participants were randomized to intervention and control based on a 70:30 ratio. In total, 774 clients participated in the trial and similar to previous studies only clients scoring above cut-off on symptoms of depression and/or anxiety were included [30]. For the present study, primarily data from participants who received PMHC were used (*n* = 459), while descriptive statistics from the control arm were only used for illustrative purposes (*n* = 215).

### Interventions

Details on the interventions are described previously [22, 30, 31], but a summary follows below.

Care in PMHC is based on cognitive behavioral therapy (CBT) [17, 22]. All therapists have received training in delivering CBT. Both low intensity care (i.e. guided self-help and psycho-educative courses) and high intensity care (individual face-to-face therapy) are offered, in stepped care variants [17, 22]. Client preferences and information from the initial assessment are used to determine care. In accordance with the official guidelines from the Norwegian Directorate of Health [17], most clients were initially offered a four-session psychoeducational course.

In the PMHC group, clients took part in a median of 5 (IQR = 4–9) treatment sessions [30]. Less than one percent received guided self-help as the primary care form. About one third had group-based psychoeducation as the primary care form, one in third had individuals CBT as the primary care form, and one in three received a combination of these care forms. Almost 80% completed treatment (defined as therapist reporting the treatment goal as fulfilled and/or having completed at least six sessions), and the median length of treatment was 9.4 weeks (IQR 4.9– 21.1) [22].

Care in the TAU potentially included all ordinary services available to the target population. Examples are follow-up by the GP, private psychologists and occupational health services. In the letter where TAU clients were informed about their allocation, they were also encouraged to contact their GP for further follow-up. References to publicly available self-help resources (internet, books) were also included.

A year after PMHC, about 1 in 4 of respondents in the PMHC group reported to have (since baseline) received additional care for their mental health problem from outside of PMHC [31]. For 12% of the participants, care had been provided by other specialist health services. In the TAU group, 50% of respondents had since baseline received care for their mental health problem, 42% from specialist services [31].

### Data collection during follow-up

**Fig. 1:**
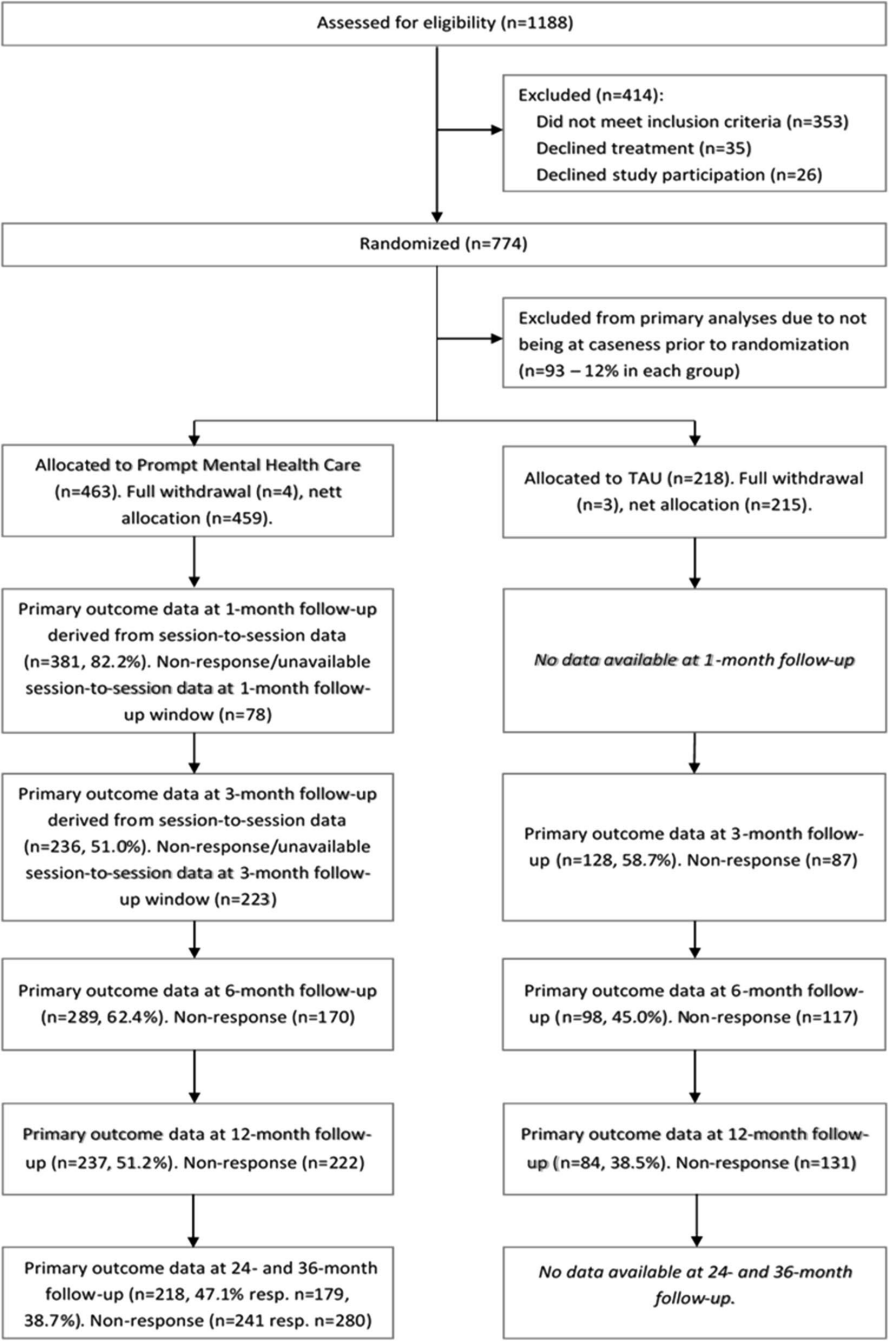
Flow diagram for participants in the RCT of Prompt Mental Health Care

Clients assigned to the PMHC group were asked to complete questionnaires before each session during the treatment, after treatment, and at 6-, 12-, 24- and 36-month follow-up. To maximize use of available data, proxies for 1.5-month follow-up (*n* = 381) and 3-month follow-up (*n* = 223) were constructed for the PMHC group based on the questionnaires that were completed prior to each session. For the 1.5-month follow-up, the last observed measurement prior to 10 weeks after baseline was used and resulted in a variable with a median time since baseline value of 6.3 weeks. For the 3-month follow-up, the last observed measurement between 10 and 14 weeks after baseline was used. For clients who terminated treatment prior to 10 weeks, the posttreatment score was carried forward to 3-month follow-up under the assumption of short-term stability, similar to the procedure that was used in earlier work [30]. The median time since baseline was 11.7 weeks. As shown in Fig. 1, primary outcome data at 24- and 36-months follow-up were available for 47% and 39% of participants. At least one follow-up measurement was available for 91% of the participants (*n* = 416) implying that there were just 43 participants with baseline data only. Clients assigned to the TAU group were asked to complete questionnaires at 3-, 6- and 12-month follow-up only [33].

### Primary outcomes

Symptoms of depression were measured using the Patient Health Questionnaire (PHQ-9) [34, 35]. This measure includes nine items based on each of the DSM-IV criteria for depression. Participants indicate how often during the last two weeks they have experienced each symptom. Response options range from 0 (“not at all”) to 3 (“nearly every day”, giving a sum score ranging from 0 to 27. Caseness was defined as PHQ ≥ 10. The PHQ has been shown to have good psychometric properties [34]. In PMHC data, Cronbach’s alpha is 0.80.

Symptoms of anxiety were measured using the Generalized Anxiety Disorder Assessment (GAD-7) [35, 36]. GAD-7 measures frequency of seven common symptoms of anxiety. Response options range from 0 (“not at all”) to 3 (“nearly every day”), giving a sum score ranging from 0 to 21. Caseness was defined as GAD ≥ 8. In addition to measuring generalized anxiety disorder [36], GAD-7 also seems to have good sensitivity and specificity for panic, social anxiety, and post-traumatic stress disorder [37]. In PMHC data, Cronbach’s alpha is 0.83.

Recovery was defined as scoring above the caseness threshold on the PHQ-9 (≥ 10) and/or GAD-7 (≥ 8) measures at the start of treatment and below the caseness threshold on both these measures at follow-up. The reliable recovery rate was calculated to account for measurement error, aligning with the procedures employed for the IAPT evaluations [19]. Using the standard deviation (SD) of the sample and Cronbach’s alpha for PHQ-9 and GAD-7, a change score of ≥ 6 was derived for PHQ-9 and ≥ 5 for GAD-7. A client was defined as reliably recovered when scoring below threshold on both measures at follow-up and showing reliable improvement on either PHQ-9 or GAD-7.

### Secondary outcomes

Functional status was measured using the Work and Social Adjustment Scale (WSAS) [38]. WSAS contains 5 items and assesses impairment due to mental health problems in five domains during the last month (0 = not impaired to 8 = severely impaired). The scale has been found to perform comparably to the PHQ-9 and GAD-7 [39]. WSAS was not measured under treatment.

Health-related quality of life (HRQL) was measured using the Norwegian version of the EQ-5D-5L [40, 41]. The paper version was used but it was largely completed electronically (we did not use a dedicated digital version of the EQ-5D-5L). In absence of a Norwegian value set, the English value set was used to calculate index scores [42]. Although the latter is suboptimal, it is in line with recommendations from the literature [43]. Index scores ranged from -0.285 (worst health state) to 1 (best health state). We also reported the index scores for item 5 on anxiety and depression as a separate outcome based on feedback from one of the reviewers. The scores on item 5 ranged from 0 (no anxiety/depression) to 0.289 (extreme anxiety/depression). Among primary care clients, HRCL as measured by the EQ-5D-5L has been found strongly associated with depression, and improves when depression is treated [44].

Positive mental wellbeing was measured using the Short Warwick Edinburgh Mental Well-Being Scale, sWEMWBS [45]. The sWEMWBS contains 7 items, all measured on a scale ranging from 1 (“none of the time”) to 5 (“all the time”). The higher scores indicate more positive mental well-being. The sWEMWBS psychometric properties are satisfactory) [46, 47], also in the PMHC setting in Norway [48].

Work participation was assessed by means of two questions, one multiresponse item about current work status and one multiresponse item about sources of income. Based on these two questions, it was determined whether participants were in full- or part-time regular work without receiving benefits or not (coded as a binary variable).

### Other outcomes

To examine relapse rates at 24- and 36-months follow-up, we included clients that started treatment with case-level depression and/or anxiety symptoms who were reliably recovered at 6-month follow-up and completed PHQ-9 and GAD-7 at 24- and 36-month follow-up (*n* = 116/106), that is 6-month to 24-month, and 6-month to 36-month relapse rates. To be counted as a relapse event, symptom scores at 24- and 36-month follow-up for at least one of the outcome measures were (1) above level for caseness and were (2) ≥ 6 (PHQ-9) or ≥ 5 (GAD-7) points greater than the symptom scores at 6-months follow-up. A similar definition was used in previous study examining relapse rates in IAPT [25].

### Statistical analyses

Basic descriptive data at baseline was reported. For all models, site (Kristiansand municipality versus Sandnes municipality) was included as a fixed effect. Multiple imputation was used to estimate (reliable) recovery rates at follow-up. In the first step, 200 datasets containing fourteen variables (PHQ-9 at 7 time points, GAD-7 at 7 time points and site) were generated using Bayesian analysis (MCMC algorithm). In the second step, (reliable) recovery was conditioned on site using robust maximum likelihood, and model estimates were used to derive (reliable) recovery rates in the PMHC group at follow-up. Model constraints were used to determine whether (reliable) recovery rates were significantly different at 24- and 36-month follow-up compared to 6-month follow-up.

Latent growth models were used to model the course of outcome measures over time. Non-linearity was initially modelled by means of quadratic and cubic slopes. Piecewise models and other time transformations were also considered (exponential, log, hyperbolic) when model fit was poor. Model fit was assessed by using the Comparative Fit Index (CFI) and the Root Mean Square Error of Approximation (RMSEA). CFI ≥ 0.95, and RMSEA ≤ 0.06 were considered indicative of good model fit [49]. For continuous outcomes, within-group effect sizes (d) were calculated by dividing the estimated change from baseline to follow-up by the estimated standard deviation at baseline. Model constraints were adopted to determine whether symptom levels were different at 24- and 36-month follow-up compared to 6-month follow-up. Robust maximum likelihood was used as estimator, providing unbiased estimates under the assumption of data missing at random (MAR) [50].

Sensitivity analyses were performed for the continuous outcome measures of depression and anxiety to examine the impact of missing data at follow-up under various missing not at random (MNAR) conditions, employing both pattern mixture and selection models [50]. These models rely on fundamentally different assumptions, i.e., pattern mixture models (PMMs) assume that outcome scores are conditional on missingness, whereas selection models assume that missingness is conditional on the observed outcomes scores. That is, the pattern mixture model stratifies the sample by missing data pattern and estimates the model separately within each pattern, while the selection model implements a regression equation in which missingness is regressed on the observed scores. The overall confidence in the results presented in this study would increase if MNAR models based on different underlying assumptions produce similar results as the original MAR model. For the pattern mixture models, we created five groups by number of available data points (0: 1–2 datapoints, 21.8%; 1: 3–4 datapoints, 25.3%; 2: 5 datapoints, 13.5%; 3: 6 datapoints, 19.8%, 4: 7 datapoints, 19.6%). A multiple group model was used to estimate the latent growth model in each group, and these results were used to calculate a weighted estimate of the growth parameters. Neighbouring case missing variable restriction was used for identification purposes. For the selection models, a binary missing data indicator was created based on the following rules: value one is assigned to the time point after the last time point an individual is observed, value missing is assigned to all time points after the value of one, and value zero is assigned to all time points before the value of one. The binary missing data indicator variable at time ‘t’ was regressed on the observed outcome scores at times ‘t’ and ‘t-1’. The regression estimates for ‘t’ and ‘t-1’ were held equal across time for identification purposes. The main analyses were conducted using Mplus version 8.7.

## Results

### Demographic characteristics

The demographic characteristics of individuals taking part in this RCT have already been published [30, 31]. In short, about 60% were women and the mean age was 34 years (SD = 12.2 SD). Over 40% reported higher education, almost 40% reported to be in regular work, and less than half were single. The therapists reported 38.3% to have depression, 19.2% to have anxiety, and 42.6% to have mixed anxiety and depression as a provisional diagnosis.

### Exposure to treatment beyond 12-month follow-up

The percentage of clients in the PMHC group that reported to have received help for their mental health problems from other services since inclusion in PMHC was 24.9%, 33.0%, and 32.3% at respectively 12-, 24- and 36-month follow-up. Additional specialist care by a psychologist/psychiatrist was received by 12.2%, 21.0%, and 20.0% of the clients at respectively 12-, 24- and 36-month follow-up. The remaining clients received help from their GP or from other services at the municipality level.

### Primary outcomes

Previous work showed that the recovery rates at 6- and 12-month follow-up were significantly higher in the PMHC group compared to the TAU group (6 m = 25.5%, 12 m = 19.3%, [31]). Analysis of the data at 24- and 36-month follow-up indicated that the estimated recovery rates in the PMHC group (RR_24m_ = 63.4%, 95%CI 57.9–69.1; RR_36m_ = 66.2%, 95%CI 60.1–72.2) remained unchanged as compared to the estimated rate at 6-month follow-up (RR_6m_ = 61.9%, 95%CI 56.7–67.1); all *p* > 0.05). A similar pattern was found for the estimated reliable recovery rates (RR_6m_ = 56.5%, 95%CI 51.1–61.9; RR_24m_ = 57.5%, 95%CI 51.7–63.4; RR_36m_ = 59.7%, 95%CI 53.4–66.1; all comparisons *p* > 0.05). Due to the high degree of missingness at 24- and 36-month follow-up, the reported estimates based on imputed data should be interpreted with some caution. For comparison’s sake, we also provide the observed recovery rates: recovery rate at 24-months = 69.1%, reliable recovery rate at 24-months = 63.4%, recovery rate at 36-months = 69.1%, reliable recovery rate at 36-months = 63.4%.

**Fig. 2:**
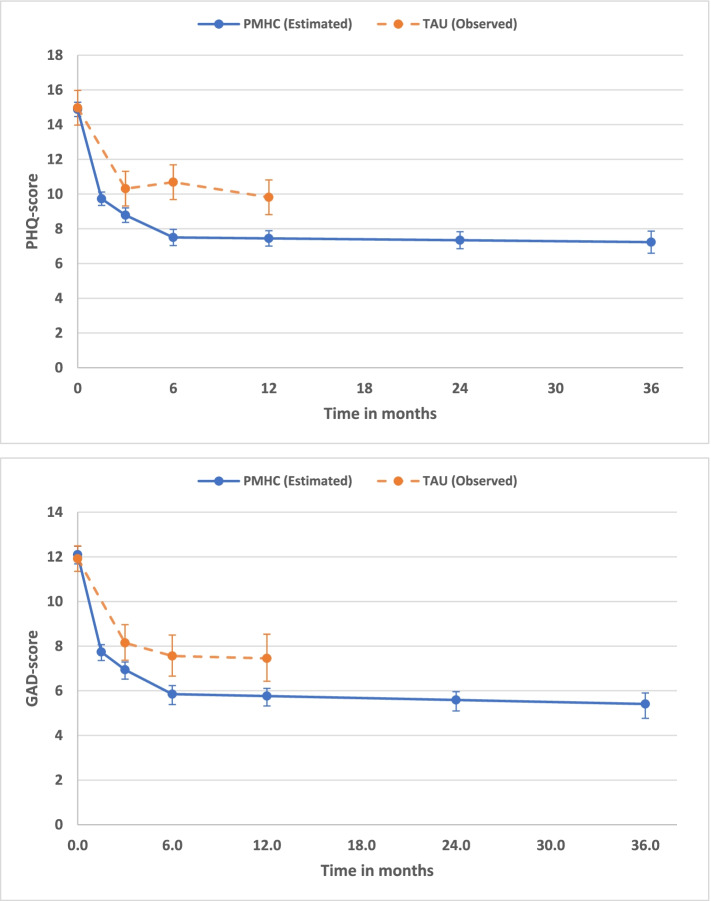
Change in mean scores of symptoms of depression (PHQ) and anxiety (GAD) from baseline up to 36-month follow-up. Observed scores in the TAU group are added for illustrative purposes

The courses of symptoms of depression and anxiety were reasonably well modelled by means of piecewise growth models with a hyperbolic time transformation for the first piece from baseline to 6-month follow-up, and a simple linear slope beyond 6-months (i.e. one intercept, two linear slopes). Fit statistics for the model with symptoms of depression as outcome were RMSEA = 0.051 and CFI = 0.948, and for the model with symptoms of anxiety as outcome RMSEA = 0.065 and CFI = 0.909. As visualized in Fig. 2, there were no statistically significant differences between symptom scores at 24- and 36-month follow-up compared to 6-month follow-up (all comparisons *p* > 0.05). The estimated linear slopes between 6-month and 36-month follow-up were -0.01 (95%CI: -0.03, 0.01) for PHQ and -0.02 (95%CI: -0.03, 0.00) for GAD. Point estimates of the within-group effect sizes from 6-month follow-up and beyond with reference to baseline varied between -1.64 and -1.70 for PHQ, and between -1.50 and -1.61 for GAD (see Table 1).

**Table 1:** Estimated means and within-group effect sizes for recipients of PMHC across continuous primary and secondary outcomes

	**Estimated means (95% CI)**	**Estimated means (95% CI)**	**Estimated means (95% CI)**	**Estimated means (95% CI)**	**Estimated means (95% CI)**	**Effect size (95% CI)**	**Effect size (95% CI)**	**Effect size (95% CI)**	**Effect size (95% CI)**
	**Baseline**	**6 months**	**12 months**	**24 months**	**36 months**	**6 months**	**12 months**	**24 months**	**36 months**
PHQ	14.88 (14.47, 15.29)	7.50 (7.03, 7.97)	7.45 (7.01, 7.89)	7.34 (6.85, 7.83)	7.23 (6.60, 7.87)	-1.64 (-1.79, -1.49)	-1.65 (-1.80, -1.50)	-1.67 (-1.83, -1.52)	-1.70 (-1.88, -1.52)
GAD	12.10 (11.72, 12.48)	5.85 (5.47, 6.23)	5.76 (5.42, 6.11)	5.59 (5.21, 5.96)	5.41 (4.91, 5.91)	-1.50 (-1.64, -1.36)	-1.52 (-1.66, -1.38)	-1.57 (-1.71, -1.42)	-1.61 (-1.78, -1.44)
WSAS	21.80 (21.09, 22.50)	13.18 (12.09, 14.26)	11.79 (10.54, 13.04)	12.04 (10.77, 13.31)	10.68 (9.29, 12.08)	-1.11 (-1.28, -0.95)	-1.29 (-1.49, -1.10)	-1.26 (-1.46, -1.07)	-1.44 (-1.65, -1.22)
EQ-5D-5L	.64 (.62, .66)	.81 (.79, .82)	.81 (.79, .84)	.81 (.79, .83)	.81 (.79, .84)	.81 (.72, .90)	.82 (.73, .90)	.83 (.74, .93)	.85 (.74, .96)
Item 5 EQ-5D-5L	.16 (.15, .17)	.08 (.08, .09)	.08 (.08, .09)	. .08 (.07, .09)	.08 (.07, .09)	-.84 (-.93, -.75)	-.85 (-.94, -.76)	-.88 (-.98, -.79)	-.91 (-1.03, -.79)
WEMWBS	18.21 (17.87, 18.56)	23.43 (22.95, 23.91)	23.68 (23.22, 24.14)	24.20 (23.69, 24.70)	24.72 (24.06, 25.37)	1.41 (1.24, 1.57)	1.47 (1.31, 1.64)	1.61 (1.43, 1.79)	1.75 (1.54, 1.97)

*Note: PHQ* Symptoms of depression*, GAD* Symptoms of anxiety*, WSAS* Functional status*, EQ-5D-5L* Health-related quality of life, *Item* 5—*EQ-5D-5L* Anxiety and Depression item*, WEMWBS* Positive mental well-being

### Secondary outcomes

The courses of health-related quality of life and positive mental well-being were modelled in the same way as symptoms of anxiety and depression and gave fit statistics of RMSEA = 0.058/ CFI = 0.942 and RMSEA = 0.053/ CFI = 0.952, respectively. The course of functional status was modelled by means of a piecewise growth curve model as well, but without a time transformation and both parts (prior and after six months) were modelled by two sets of linear and quadratic slopes. As both parts were only based on three timepoints each, the quadratic slope variances were restricted to zero for identification purposes (RMSEA = 0.028/ CFI = 0.994). Due to issues with multicollinearity, measurements at 1.5- and 3-month follow-up were not included to determine the course of work participation. The latter outcome was subsequently modelled by a simple linear growth model (intercept, linear slope) with a log-transformed time scale (RMSEA = 0.026/ CFI = 0.997).

For functional status, the WSAS score continued to improve with a mean change score of 1.14 at 24-month follow-up (*p* = 0.09), and a mean change score of 2.50 at 36-month follow-up (*p* < 0.001), all compared to 6-month follow-up. Positive mental well-being also continued to improve at 24-month follow-up with a mean change score of 0.77 (*p* < 0.001), and at 36-month follow-up with a mean change score of 1.29 (*p* < 0.001). For health-related quality of life, the estimated change at 24- and 36-month follow-up as compared to 6-month follow-up was not statistically significant (both *p* > 0.05). This was also the case when examining item 5 of the EQ-5D-5L separately. See Table 1 for details on estimated means and within-group effect sizes across measurement occasions.

In previous work, we did not find evidence for an effect of PMHC on work participation up to 12-month follow-up because a similar increase in work participation was observed in both the intervention and control group [31]. The data at 24- and 36-month follow-up indicated a further increase in work participation among participants in the PMHC group. The estimated proportion of participants in full- or part-time regular work went from 37.4% at baseline to 48.7%, 59.4% and 62.2% at respectively 6-, 24-, and 36-months follow-up, and the increase at 24- and 36-month follow-up compared to the level at 6-month follow-up was statistically significant (both *p* < 0.05). As the latter two measurement points were purely observational, the increases cannot simply be attributed to the effect of the intervention, as is true for all outcomes in this study.

### Other outcomes

The relapse rate in the PMHC group was 9.5% (11/116; 95% CI 5.2 to 15.5) from 6-month to 24-month follow-up and 14.2% (15/106; 95% CI 7.5 to 20.8) from 6-month to 36-month follow-up.

### Sensitivity analyses

**Fig. 3:**
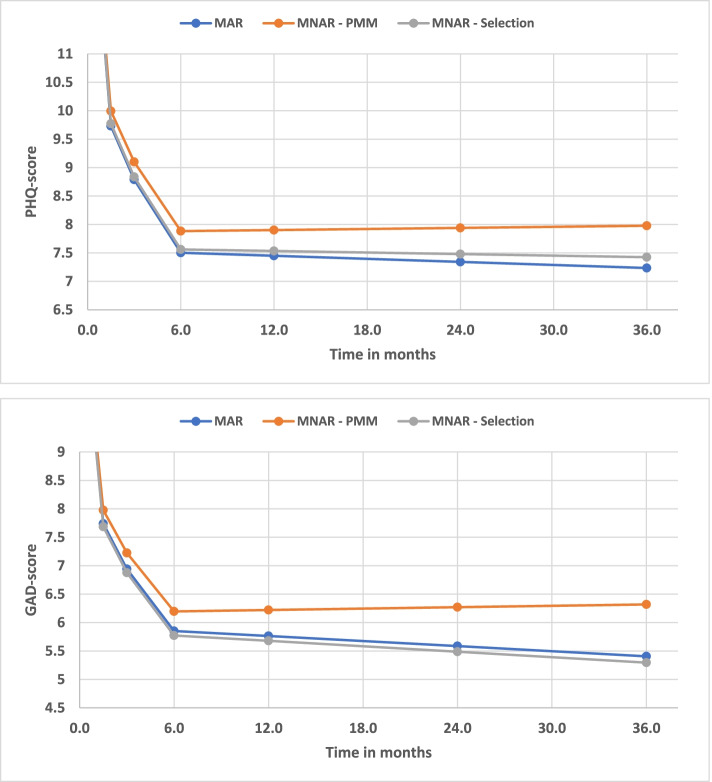
Change in mean scores of symptoms of depression (PHQ) and anxiety (GAD) in the PMHC group across different missing data assumption scenarios. Note that the range of the y-axis has been restricted, which enlarges the difference between the models

The results of the sensitivity analyses are shown in Fig. 3 and indicates that the maintained improvements observed at the 24- and 36-month follow-up in terms of symptoms of anxiety and depression also hold under certain MNAR-assumptions. Therefore, the substantial level of attrition may not necessarily have that much impact on the inference of this study. The within-group effect sizes under the MNAR conditions varied between -1.54 and -1.66 for PHQ, and between -1.39 and -1.64 for GAD at long-term follow-up.

## Discussion

Previous research has suggested that PMHC is an effective treatment for people suffering from anxiety and mild-to-moderate depression, and that this effect lasts up to at least 12-month follow-up. This current study adds to this by demonstrating that the observed symptom improvements are maintained or further improve at 24- and 36-month follow-up for those assigned to the PMHC treatment condition. Compared to the change observed between baseline and 6-month follow-up, there was no significant additional change at 24- and 36-month follow-up for symptoms of anxiety and depression, (reliable) recovery rate, and health-related quality of life. For functional status, positive mental well-being, and work participation, further improvements were noted at long-term follow-up. The relapse rate at 24-month follow-up was similar to the rate at 12-month follow-up, while there was a small increase at 36-month follow-up. Results were similar also after considering the possibility of data missing not at random, using pattern mixture and selection models.

Very few studies have so far examined the long-term effectiveness of CBT in comparable settings like IAPT and PMHC. Von Brachel et al. [51] conducted a retrospective follow-up study in 263 former outpatients who were treated for a variety of common mental health problems. The average follow-up time was 8 years and they reported a within-group effect size of 0.92 for symptoms of depression and 0.75 for psychological distress. These effect sizes were lower as compared to the ones found in our study. This may partly be explained by differences in follow-up times, but also by the fact that treatment delivery in PMHC was done in a study setting, which is known to be associated with larger effect sizes [52]. The latter effect cannot be excluded, even though the RCT of PMHC was designed to be as practice-near as possible and should therefore not be compared to a typical efficacy study either [22]. In general, there is a paucity of studies that have examined the long-term effectiveness of CBT on anxiety and depression [53, 54], and as such our study aids to fill this existing knowledge gap. It is encouraging that the effect sizes at long-term follow-up that we found were relatively large as compared to the average post-treatment effect sizes obtained in meta-analyses on the effectiveness of CBT for anxiety [55] and depression [56]. This is all the more true giving the relatively low treatment intensity and short treatment duration of PMHC. Also relapse rates remained relatively low at long-term follow-up and were not higher than observed in other studies in the shorter term [25, 53].

For three outcomes, a further improvement since 6-month follow-up was observed, most notably for functional status and work participation. Although improvements beyond treatment termination is not uncommon [51], it’s not unexpected that this was observed in these two outcomes as improvements in function often lag improvements in symptoms [57, 58]. Given the lack of a control group at 24- and 36-month follow-up, the effect of PMHC treatment on work participation remains inconclusive though [30, 31].

### Strengths and Limitations

The main strengths of this study are the long follow-up time, the relatively large sample size, the use of validated and reliable questionnaires, the use of modern statistical techniques that ensure that all study participants are included in the analyses despite non-response at follow-up, and the consideration of models under MNAR conditions.

The most important sources of potential bias were lack of long-term follow-up data in the control group and substantial attrition. With regard to the latter, all sensitivity analyses pointed in the same direction and had effect sizes of similar magnitude suggesting that the impact of attrition may not be that large. On the other hand, we only test some of all potential MNAR scenarios, and it’s important to note that all these scenarios are based on untestable assumptions. Given the level of attrition in the present study, our findings should therefore nevertheless be interpreted with caution.

## Conclusion

The current study suggests that PMHC can produce long-lasting improvements in symptoms, function, mental well-being and health-related quality of life. These are all aspects central to the individual and to the economic evaluation of care. As such, the study adds further evidence that this version of IAPT can be considered a viable supplement to existing health services.

## Data Availability

The datasets analyzed during the current study are not publicly available due to ethical restrictions and personal data protection but are available from the corresponding author on reasonable request.

## Abbreviations

CBT: Cognitive behavioral therapy
CFI: Comparative fit index
CI: Confidence interval
DSM: Diagnostic and statistical manual of mental disorders
EQ5D: EuroQol- 5 dimension
GAD: Generalized anxiety disorder
GP: General practitioner
HRQL: Health-related quality of life
IAPT: Improving access to psychological therapies
IQR: Inter quartile range
MAR: Missing at random
MCMC: Markov chain monte carlo
MNAR: Missing not at random
NSD: Norwegian center for research data
PHQ: Patient health questionnaire
PMHC: Prompt mental health care
PMM: Pattern mixture model
RMSEA: Root mean square error of approximation
RCT: Randomized controlled trial
SD: Standard deviation
TAU: Treatment as usual
WEMWBS: Warwick edinburgh mental well-being scale
WSAS: Work and social adjustment scale
